# Potent Anti-Inflammatory Activity of Pyrenocine A Isolated from the Marine-Derived Fungus *Penicillium paxilli* Ma(G)K

**DOI:** 10.1155/2014/767061

**Published:** 2014-01-19

**Authors:** Thaís Regina Toledo, Naiara N. Dejani, Luis Gustavo Silva Monnazzi, Miriam H. Kossuga, Roberto G. S. Berlinck, Lara D. Sette, Alexandra I. Medeiros

**Affiliations:** ^1^Departamento de Ciências Biológicas, Faculdade de Ciências Farmacêuticas, Universidade Estadual Paulista “Júlio de Mesquita Filho”, 14801-902 Araraquara, SP, Brazil; ^2^Faculdade de Medicina Ribeirão Preto, Universidade de São Paulo, 14049-900 Ribeirão Preto, SP, Brazil; ^3^Instituto de Química de São Carlos, Universidade de São Paulo, CP 780, 13560-970 São Carlos, SP, Brazil; ^4^Departamento de Bioquímica e Microbiologia, Instituto de Biociências, Universidade Estadual Paulista “Júlio de Mesquita Filho”, 13506-900 Rio Claro, SP, Brazil

## Abstract

Very little is known about the immunomodulatory potential of secondary metabolites isolated from marine microorganisms. In the present study, we characterized pyrenocine A, which is produced by the marine-derived fungus *Penicillium paxilli* Ma(G)K and possesses anti-inflammatory activity. Pyrenocine A was able to suppress, both pretreatment and posttreatment, the LPS-induced activation of macrophages via the inhibition of nitrite production and the synthesis of inflammatory cytokines and PGE2. Pyrenocine A also exhibited anti-inflammatory effects on the expression of receptors directly related to cell migration (Mac-1) as well as costimulatory molecules involved in lymphocyte activation (B7.1). Nitrite production was inhibited by pyrenocine A in macrophages stimulated with CpG but not Poly I:C, suggesting that pyrenocine A acts through the MyD88-dependent intracellular signaling pathway. Moreover, pyrenocine A is also able to inhibit the expression of genes related to NF**κ**B-mediated signal transduction on macrophages stimulated by LPS. Our results indicate that pyrenocine A has promissory anti-inflammatory properties and additional experiments are necessary to confirm this finding *in vivo* model.

## 1. Introduction

Inflammation is a complex host immune system response to stimuli such as microbial infection, endotoxin exposure, burns, and tissue injury. Resident macrophages are the first line of defense against insults and play a key role in the development of inflammation [1, 2]. These phagocytes have a variety of receptors on their surface membrane, termed Pattern Recognition Receptors (PRRs), which facilitate interactions with many molecules present on pathogens, known as PAMPs (pathogen-associated molecular patterns). PRRs such as TLRs (Toll-like receptors) interact with PAMPs that are present on bacteria, viruses, parasites, and fungi, and these interactions play a key role in the activation of phagocytic cells. Lipopolysaccharide (LPS), which is produced by Gram-negative bacteria, binds with a TLR complex composed of CD14/LBP/TLR [1, 2], resulting in the activation of a complex biochemical cascade that promotes the recruitment of MyD88, the activation of protein kinases such as IRAK, recruitment of the adaptor protein TRAF6, and the subsequent activation of NF*κ*B and AP-1 in the nucleus [3]. Cytosolic I*κ*B is only phosphorylated after stimulation by lipopolysaccharide (LPS), resulting in the dissociation of the I*κ*B complex and the translocation of NF*κ*B into the nucleus, permitting interaction with the promoter regions of various genes that encode proinflammatory mediators [4]. Activation of these genes results in the excessive production of inflammatory cytokines including TNF-*α*, interleukin-1 (IL-1), and IL-6 and other inflammatory mediators such as nitric oxide (NO) and lipid mediators, all of which play a deleterious role in acute and chronic inflammatory diseases such as atherosclerosis and arthritis [5]. In particular, prostaglandin E2 (PGE2) is a major inflammatory lipid mediator involved in the pathogenesis of chronic inflammatory diseases such as rheumatoid arthritis [6]. PGE2 synthesis occurs when there are disturbances in the cell membrane, leading to increased intracellular calcium and the translocation of cytosolic phospholipase A2 (cPLA2) to the nuclear membrane, followed by the release of arachidonic acid (AA) from phospholipids in the nuclear membrane. The released AA can be converted into PGH2 in the presence of COX-1 (an enzyme constitutively expressed in different cell types) and COX-2 (an inducible enzyme) and then through the action of various prostaglandin synthases to generate different classes of PGs. PGE2 is a key lipid mediator synthesized by macrophages and other cell types in the presence of LPS [7]. In addition to promoting edema, PGE2 can indirectly act to modulate the synthesis of different cytokines and chemokines [8, 9]. PGE2 plays a role in the development of inflammation at sites of tissue injury by interacting with EP1/EP3 and therefore is a prime target for potential anti-inflammatory drugs in the treatment of acute and chronic inflammatory diseases [10].

Marine microorganisms are unique biological sources of potently active secondary metabolites, which have been investigated over the last 20 years with the goal of discovering new drugs [11–13]. Marine fungi are considered to be particularly rich metabolic sources of active natural products [12–16]. Recently, we isolated pyrenocine A from the marine-derived fungus *Penicillium paxilli *Ma(G)K. Pyrenocine A was previously reported to be an antimalarial and a cytotoxic agent that was active against KB, BC-1, and Vero cancer cells [17]. However, there have been no reports describing the immunoregulatory activity of pyrenocine A.

Given the importance of identifying potential new anti-inflammatory drugs, the aim of this study was to investigate the pretreatment and posttreatment anti-inflammatory potential of pyrenocine A and to elucidate the possible mechanisms through which this fungal metabolite can act on immune cells.

## 2. Material and Methods

### 2.1. Isolation and Identification of Pyrenocine A

Pyrenocine A was previously obtained from the growth culture medium of the marine-derived fungus *P. paxilli *Ma(G)K, which was isolated from the sponge *Mycale angulosa *[18]. Briefly, *P. paxilli *Ma(G)K was inoculated in 500 mL Schott flasks, each containing 200 mL of 2% malt extract medium. Fungal growth occurred with shaking (100 rpm) at 25°C for seven days. At the end of the growth period, 200 mL of ethyl acetate (EtOAc) was added to each flask. The culture media mixture containing mycelia and EtOAc was left shaking at 100 rpm for 24 h at 25°C. Then, the mixture was filtered through Celite. The EtOAc fraction was separated by liquid-liquid partitioning. Evaporation of the EtOAc fraction yielded 400 mg of an extract, which was separated by chromatography on a cyanopropyl-bonded silica-gel cartridge (2 g; eluents: 100% CH_2_Cl_2_, 100% EtOAc, and 100% MeOH). The CH_2_Cl_2_ fraction (130 mg) was separated by HPLC using a C_18_ reversed phase column Inertsil ODS-3 (4.6 × 250 mm; 5 *μ*m) with a gradient of 1 : 1 MeOH : MeCN in H_2_O plus 0.1% formic acid (flow rate: 1.0 mL/min; detection: UV absorption at *λ*_max_ 254 nm). Pyrenocine A (42.7 mg) was identified by analyzing the spectroscopic data and comparing them with the data in the literature [18].

### 2.2. Cell Line Maintenance

The RAW 264.7 cell line, which was used in all *in vitro* experiments, was obtained from the Rio de Janeiro Cell Bank (BCRJ/UFRJ). The RAW 264.7 cell line is a mouse leukemic monocyte-macrophage cell line. For cytotoxicity assays and anti-inflammatory activity assays, cells were thawed and expanded into cell culture flasks in DMEM containing 10% FBS (fetal bovine serum) and gentamicin (1 : 1000) at 37°C in an incubator with a humidified atmosphere containing 5% CO_2_, following the American Type Culture Collection (ATCC) guidelines.

### 2.3. Cell Stimulation: Pretreatment and Posttreatment Protocol, Cytotoxicity Assays, and Cell Death

RAW 264.7 cells (5 × 10^ 4^) were distributed in 96-well plates and used for pretreatment and posttreatment assays. Lyophilized pyrenocine A was reconstituted in DMSO and then diluted in DMEM-I culture medium. Pretreatment protocol: pyrenocine A (3.75 then to 0.11 *μ*M) was added to the cell culture for 2 h, and stimuli with LPS, CpG or Poly I:C (1 *μ*g/mL) for 18 h at 37°C. Posttreatment protocol: cells were incubated with LPS, CpG, or Poly I:C (1 *μ*g/mL) for 2 h, and then pyrenocine A was added to the cell culture (3.75 to 0.11 *μ*M) for 18 h at 37°C. The supernatants were collected and stored at −80°C until quantification of soluble mediators was performed.

The cytotoxicity was evaluated by MTT and Alamar Blue assay and cells death by Annexin V/PI (BD Biosciences) as manufacturer's recommendation.

### 2.4. Quantification of Nitrite

Nitrite levels in the culture supernatant samples were measured with Griess reagent as previously described [19]. The absorbance of the samples was measured in a microplate reader with a 540 nm filter (Biotek, model 960).

### 2.5. Quantification of Cytokines and PGE2 Levels

Cytokine production by macrophages was quantitated from culture supernatants using an ELISA reader. Levels of IL-1*β*, IL-6, IL-10, IL-12, and TNF-*α* were determined using specific antibodies (purified and biotinylated) and recombinant cytokine standards following the manufacturer's instructions (Opteia B & D Systems, MN and PharMingen, San Diego, CA). PGE2 was measured in culture supernatants using the EIA kit for PGE2 with a monoclonal antibody according to the manufacturer's instructions (Cayman Chemical Company).

### 2.6. Flow Cytometry

The expression of surface receptors (CD11b/CD18 and B7.1, B7.2) was determined after treating RAW 264.7 cells with pyrenocine A using both the pretreatment and posttreatment procedures as previously described. All samples were analyzed using a FACSCanto flow cytometer (Becton Dickinson and San Jose, CA) and FACS DIVA software.

### 2.7. RNA Isolation and Quantitative RT-PCR

Total RNA was extracted and purified using silica-based spin columns (Qiagen RNeasy Mini Kit) following the manufacturer's instructions. The isolated total RNA (5 *μ*g) was reverse-transcribed using the High Capacity cDNA Reverse Transcription Kit (cat. number: 4391526, Applied Biosystems). Quantitative real-time PCR (qRT-PCR) was performed with Applied Biosystems 7500 Real Time PCR equipment. Selected target genes were measured in a 96-well (TaqMan express plate, custom 2 × 48) TaqMan NF*κ*B signaling Target PCR Custom Array Plate (Applied Biosystems) as described in the manufacturer's protocol. The resulting data from three independent samples were analyzed based on the ΔCt to statistical analysis and 2^(−ΔΔCt)^ method to relative quantification (RQ) to demonstrate the variation in gene expression [20] using the reference endogenous gene *Gapdh* for normalization. Statistical significance was calculated using the two-tailed Student's *t*-test. *P* values of less than 0.05 were considered significant.

### 2.8. Statistical Analysis

The results are presented as the mean ± SEM of at least three individual experiments performed in quadruplicate (PGE2, nitrite, cytokines). Statistical analyses were performed with GraphPad Prism Instat-4. For comparisons between three or more experimental groups, ANOVA was applied followed by the Bonferroni multiple comparisons test. RT-PCR statistical significance was calculated using the two-tailed Student's *t* test. The results were considered statistically significant at *P* ≤ 0.05.

## 3. Results

### 3.1. Effect of Pyrenocine A on Cell Viability and NO Inhibition

**Figure 1:**
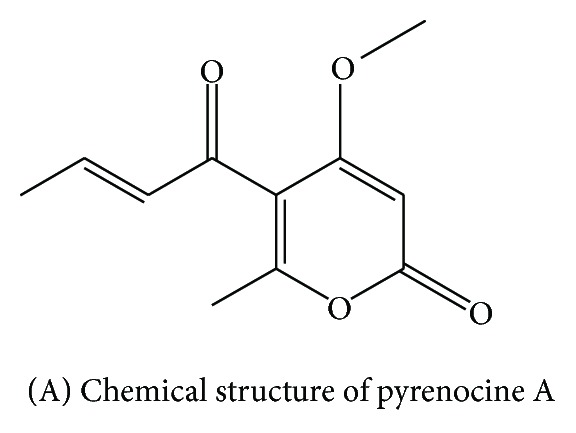
Structure of pyrenocine A produced in culture by the marine-derived fungus *Penicillium paxilli *Ma(G)K.

Pyrenocine A (Figure 1) was isolated and identified as previously described [18]. The cytotoxicity of pyrenocine A in RAW 264.7 cells was evaluated in the presence of LPS using both the pretreatment and posttreatment protocols. Our results demonstrated that, regardless of the protocol used, treatment with pyrenocine A at concentrations below 3.75 *μ*M resulted in 100% cell viability. Cells treated with pyrenocine A at 7.5 *μ*M showed a modest decrease in cell viability (<3%) (data not shown). To minimize any false negative results due to cell death rather than the anti-inflammatory effects of pyrenocine A, we used concentrations of pyrenocine A that resulted in 100% cell viability in subsequent assays. The cytotoxicity of pyrenocine A in RAW 264.7 cells was also evaluated by Alamar Blue assay (supplemental data, Figure 1(a)) and cells death by Annexin/Propidium iodide (supplemental data, Figure 1(b) in supplementary material available online at http://dx.doi.org/10.1155/2014/767061). In both assays we demonstrate that pyrenocine A alone did not induce cells death and confirm by other methods that the concentrations of pyrenocine A used in all experiments there are low or any cytotoxicity.

**Figure 2:**
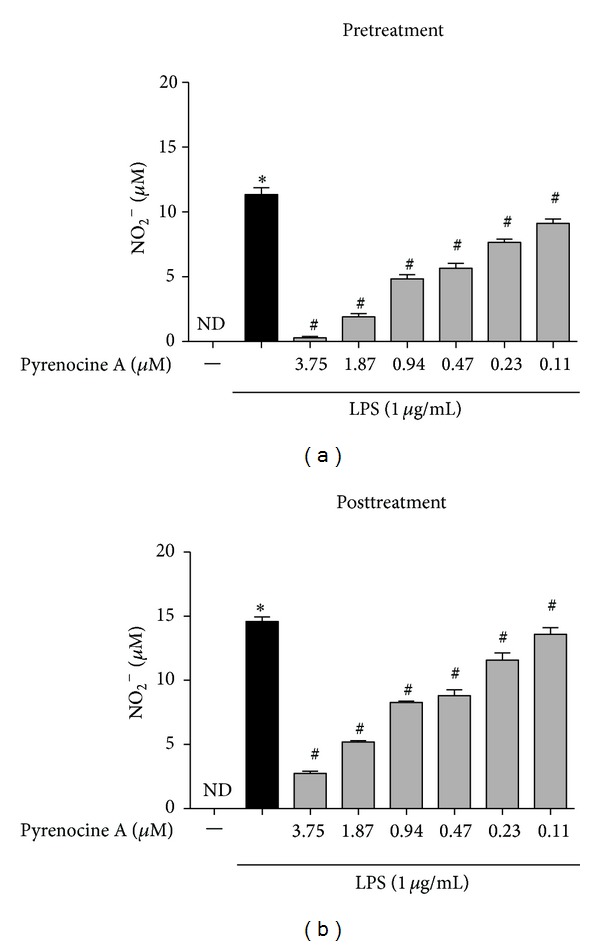
Anti-inflammatory effects of pyrenocine A on the production of NO. Pretreatment procedure: cells were pretreated with different concentrations of pyrenocine A (3.75–0.11 *μ*M) for 2 h and then stimulated with LPS (1 *μ*g/mL) during 18 h. Posttreatment procedure: cells were stimulated with LPS (1 *μ*g/mL) for 2 h and then pyrenocine A was added (3.75–0.11 *μ*M) during 18 h. Afterwards, the supernatants were collected, and nitrite concentrations were determined using Griess reagent. Results indicate the mean ± SEM for six independent experiments performed in quadruplicate for each condition. *P < 0.05 versus medium (control), ^#^ *P* < 0.05 versus LPS (ANOVA followed by Bonferroni). ND = not detectable.

Subsequently, we evaluated the anti-inflammatory potential of pyrenocine A by measuring the production of nitric oxide (NO). Stimulation of macrophages with LPS induces high levels of NO production compared to unstimulated cells. Using both protocols, pretreatment and posttreatment, we verified that pyrenocine A was able to inhibit the synthesis of NO in a concentration-dependent manner in comparison to macrophages stimulated with LPS (Figure 2). The anti-inflammatory effects of pyrenocine A on NO synthesis were evident even at low concentrations and were more pronounced with the posttreatment protocol (Figure 2). The treatment of pyrenocine A alone has no stimulatory effect on RAW 264.7 cells; the amount of NO produced is the same as untreated cells (data not shown).

### 3.2. Pyrenocine A Can Inhibit TNF-*α* Production by Macrophages

**Figure 3:**
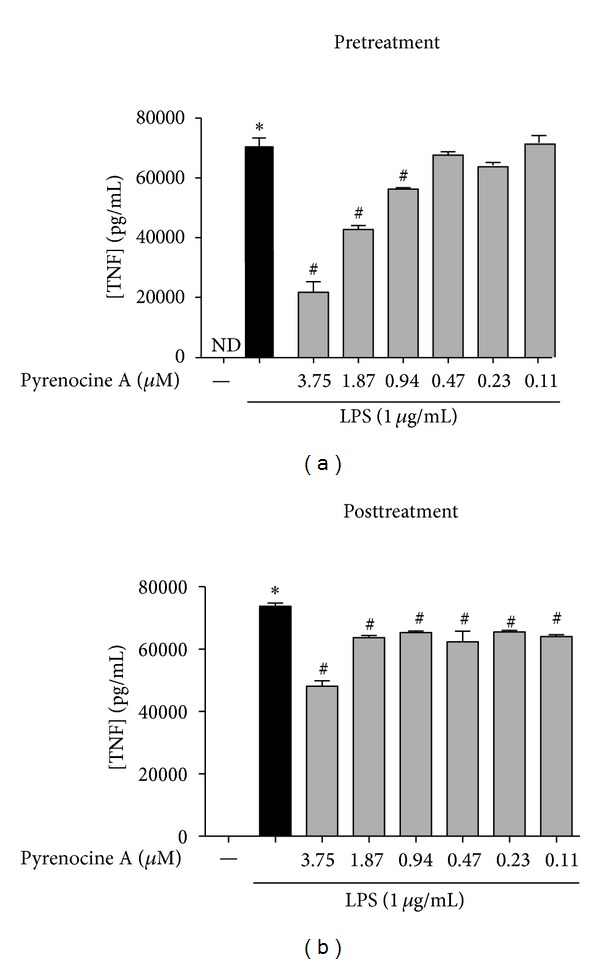
Anti-inflammatory effects of pyrenocine A on the production of TNF-*α*. (a) Pretreatment procedure: cells were pretreated with different concentrations of pyrenocine A (3.75–0.11 *μ*M) for 2 h and then stimulated with LPS (1 *μ*g/mL) during 18 h. (b) Posttreatment procedure: cells were stimulated with LPS (1 *μ*g/mL) for 2 h and then pyrenocine A (3.75–0.11 *μ*M) was added during 18 h. The supernatants were collected, and cytokine quantification was performed using an ELISA system. Results correspond to the mean ± SEM of six independent experiments performed in quadruplicate for each condition. *P < 0.05 versus medium (control), ^#^ *P* < 0.05 versus LPS (ANOVA followed by Bonferroni). ND = not detectable.

Next, we evaluated whether pyrenocine A was also able to modulate cytokine production using both experimental approaches. As shown in Figure 3, macrophages secrete high levels of TNF-*α* in the presence of LPS compared to unstimulated cells (Figure 3). LPS-induced synthesis of these inflammatory cytokines was inhibited by pyrenocine A in a concentration-dependent manner with both the pretreatment and posttreatment protocols. The inhibitory effects of pyrenocine A on TNF-*α* synthesis were more evident when the pretreatment procedure was employed (Figure 3). However, treatment of macrophages with pyrenocine A in the presence of LPS did not modulate the synthesis of other inflammatory cytokines, including IL-1*β*, IL-6, and IL-12, and had low effect on the synthesis of IL-10 (data not shown).

### 3.3. Pyrenocine A Inhibits PGE2 Synthesis

**Figure 4:**
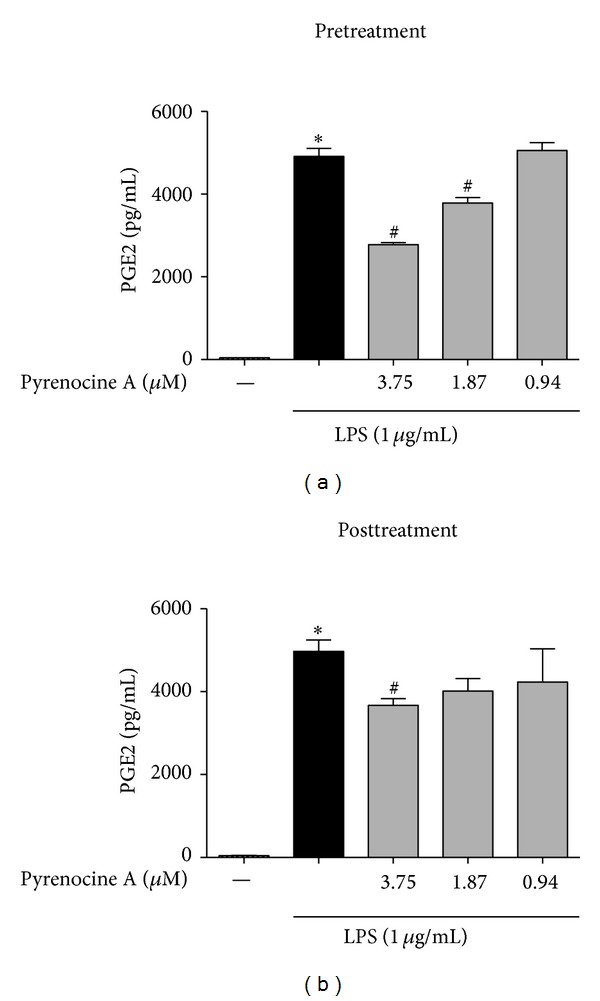
Anti-inflammatory effects of pyrenocine A on PGE2 production by macrophages. (a) Pretreatment procedure: cells were pretreated with different concentrations of pyrenocine A (3.75–0.94 *μ*M) for 2 h and then stimulated with LPS (1 *μ*g/mL) during 18 h. (b) Posttreatment procedure: cells were stimulated with LPS (1 *μ*g/mL) for 2 h and then pyrenocine A (3.75–0.94 *μ*M) was added during 18 h. The supernatants were collected, and the production of PGE2 was evaluated. The procedures were performed in triplicate.

The stimulation of macrophages with LPS induces the synthesis of lipid mediators such as PGE2, a major lipid inflammatory mediator involved in the pathogenesis of chronic inflammatory diseases such as rheumatoid arthritis [6]. Therefore, in addition to examining inflammatory cytokines and NO, we evaluated the ability of pyrenocine A to modulate the expression of PGE2 synthesis in macrophages stimulated with LPS. As shown in Figure 4, LPS stimulation induced the synthesis of high concentrations of PGE2 compared to unstimulated cells. Treatment with pyrenocine A using the pretreatment procedure inhibited the synthesis of PGE2 by approximately 30 to 50% compared to LPS alone (Figure 4(a)) and at higher concentration of pyrenocine A during the posttreatment (Figure 4(b)).

### 3.4. Inhibition of Cell Surface Receptors Involved in Cellular Adhesion and Activation by Pyrenocine A

**Figure 5:**
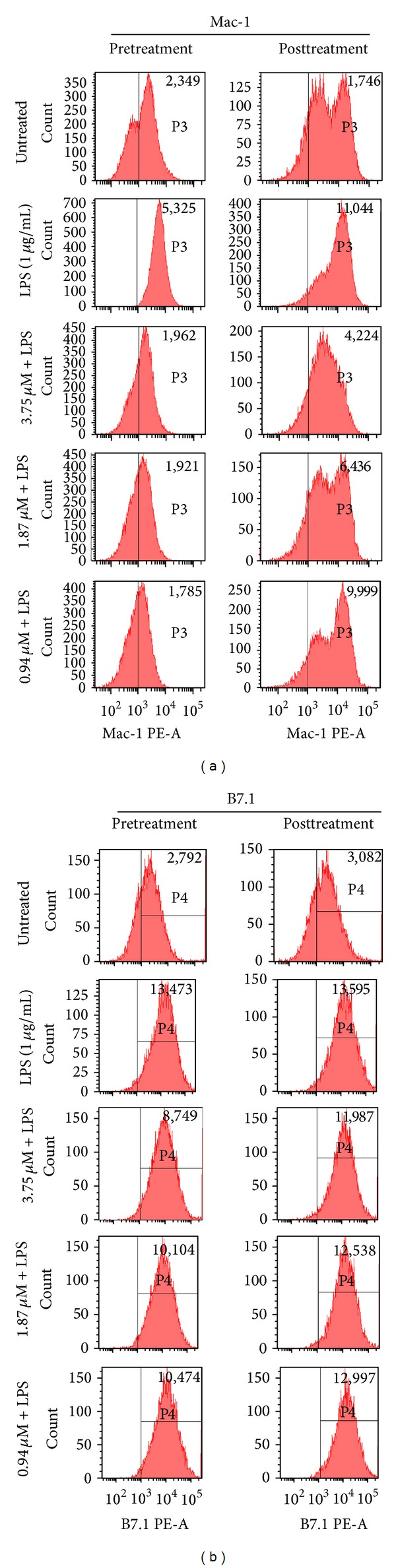
Effects of pyrenocine A on the expression of Mac-1 and B7.2 by macrophages. Pretreatment procedure: cells were pretreated with different concentrations of pyrenocine A (3.75–0.94 *μ*M) for 2 h and then stimulated with LPS (1 *μ*g/mL) during 18 h. (b) Posttreatment procedure: cells were stimulated with LPS (1 *μ*g/mL) for 2 h and then pyrenocine A was added (3.75–0.94 *μ*M) during 18 h. The expression of surface receptors such as Mac-1 and B7.1 was evaluated by MIF and by measuring the percentage of positive cells, respectively. All samples were analyzed using flow cytometry and a FACSCanto machine with BD FACSDiva software. Results are representative of three independent experiments.

During inflammation, surface molecules such as Mac-1 (CD11b/CD18) and B7.1/B7.2 play important roles in the migration and activation of macrophages [21, 22]. We next evaluated whether pyrenocine A was able to modulate the expression of this molecules in macrophages in the presence of an inflammatory stimulus (LPS). LPS stimulation increased the expression of Mac-1 and B7.1 compared to unstimulated cells (Figure 5). Treatment of macrophages with pyrenocine A with both the pretreatment and posttreatment procedures diminished the amount of B7.1 and Mac-1 on the cell surface measured by median intensity fluorescence (MIF) compared to macrophages stimulated with LPS alone. As shown in Figure 5(a), the pretreatment protocol with pyrenocine A inhibited the expression of Mac-1 at levels similar to unstimulated cells (without LPS). However, treatment with pyrenocine A in the presence of LPS resulted in only a modest inhibition of B7.2 expression on the surface of macrophages (data not shown).

### 3.5. The Suppressive Effects of Pyrenocine A Are Dependent on MyD88

**Figure 6:**
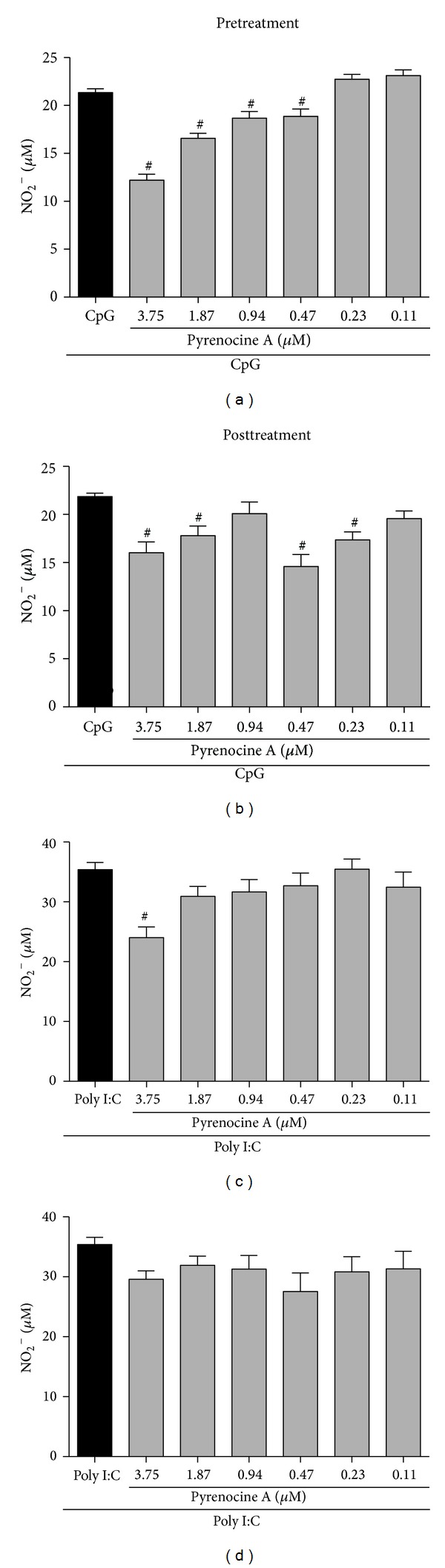
Anti-inflammatory effects of pyrenocine A by TLR9/MyD88 pathway. Pretreatment procedure: cells were pretreated with different concentrations of pyrenocine A (3.75–0.11 *μ*M) for 2 h and then stimulated with CpG (1 *μ*g/mL) during 18 h (a) or Poly I:C (1 *μ*g/mL) (c). Posttreatment procedure: cells were stimulated with CpG (1 *μ*g/mL) (b) or Poly I:C (1 *μ*g/mL) (d) for 2 h and then pyrenocine A (3.75–0.11 *μ*M) was added during 18 h. The supernatants were collected to quantify NO. Results correspond to the mean ± SEM of three independent experiments performed in quintuplicate for each condition. ^#^ *P* < 0.05 versus CpG or Poly I:C alone (ANOVA).

LPS interacts with the CD14/LBP/TLR4 complex, resulting in the activation of MyDd88-dependent and MyDd88-independent pathways within a complex biochemical cascade [1, 2]. To evaluate whether pyrenocine A exerts its anti-inflammatory effects through the MyDd88-dependent and MyDd88-independent pathways, we stimulated macrophages with two agonists: a TLR3 ligand (Poly I:C) and a TLR9 ligand (CpG-ODNs). Poly I:C promotes TLR3 activation via MyD88-independent pathway (by TRIF adapter molecule), while the binding of CpG to TLR9 induces MyD88-dependent activation. Then, we evaluate whether pyrenocine A exerts its anti-inflammatory effects by MyD88-independent or MyD88-dependent pathway using both agonists (Poly I:C and CpG-ODNs) as tools to suggest it. In the presence of CpG, the pretreatment and posttreatment with pyrenocine A on macrophages inhibited the synthesis of NO in a concentration-dependent manner when compared with CpG treatment alone (Figures 6(a) and 6(b)). However, in both pre- and posttreatment protocols, pyrenocine A was not able to inhibit NO synthesis when macrophages were stimulated with Poly I:C (Figures 6(c) and 6(d)).

### 3.6. Effects of Pyrenocine A on NF-*κ*B-Dependent Gene Expression

**Figure 7:**
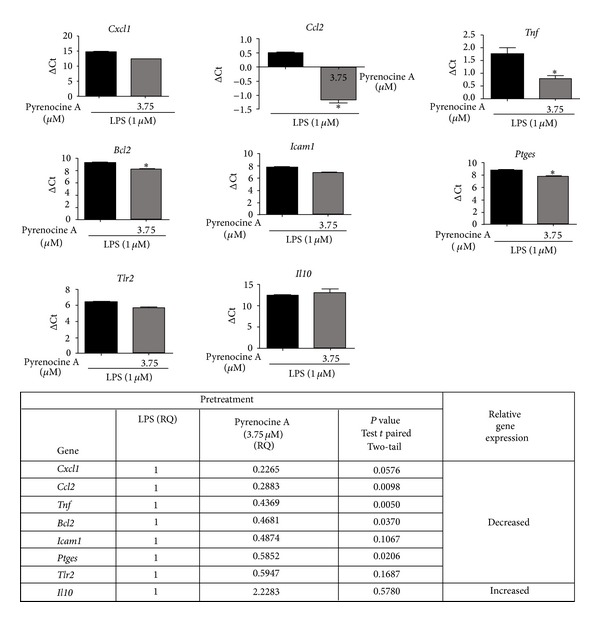
Evaluation of expression of genes related to NF*κ*B-mediated transcription-pretreatment procedure. Cells were pretreated with pyrenocine A (3.75 *μ*M) for 2 h and then stimulated with LPS (1 *μ*g/mL) during 18 h. PCR array plates were purchased from Life Technologies. The results are presented as logΔCt to statistical analysis (test *t*  *P < 0.05) and RQ (relative quantification) to show the variation in gene expression (less than 0.6 was considered decreased and more than 1.4 increased). The results show the mean and standard deviation of 3 independent experiments. *P < 0.05.

**Figure 8:**
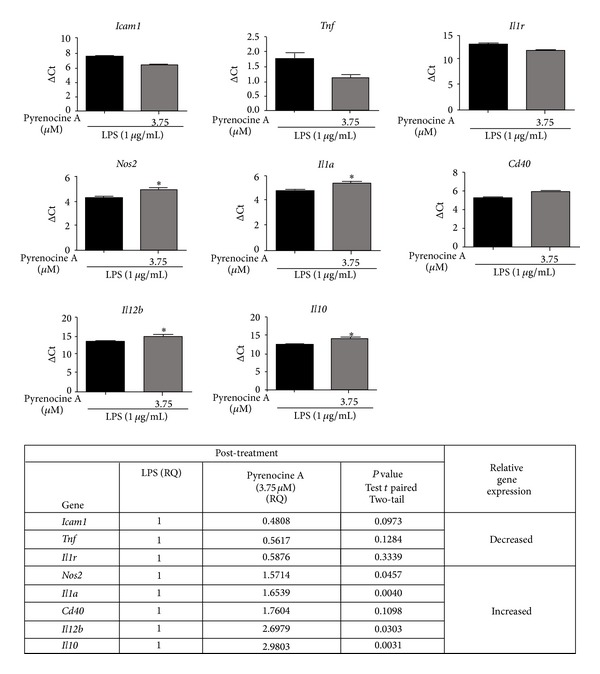
Evaluation of expression of genes related to NF*κ*B-mediated transcription-PostTreatment procedure. Cells were stimulated with LPS (1 *μ*g/mL) for 2 h and then pyrenocine A was added (3.75 *μ*M) during 18 h. PCR array plates were purchased from Life Technologies. The results are presented as logΔCt to statistical analysis (Test *t*  *P < 0.05) and RQ (relative quantification) to show the variation in gene expression (less than 0.6 was considered decreased and more than 1.4 increased). The results show the mean and standard deviation of 3 independent experiments. *P < 0.05.

We evaluated the expression of 43 genes, including cytokines, chemokines, or their receptors and components of the immune response, that are activated by NF-*κ*B signaling in macrophages after treatment with high concentration of pyrenocine A using pretreatment and posttreatment protocols, in the presence of LPS. Gene expression was considered to be downregulated when the relative quantification (RQ) was less than 0.6 and upregulated when the RQ was more than 1.4 compared to the LPS group in the absence of pyrenocine A. The RQ was considered unchanged when the value was between 0.6 and 1.4. For most of the genes tested, there were no significant (*P* value ≤0.05) differences in the mRNA levels (data not shown). However, the mRNA levels of the proinflammatory cytokine *Tnf *decreased significantly with the pretreatment procedure (Figure 7). Pyrenocine A also may exert anti-inflammatory effects by decreasing a specific mRNA of factors, such as *Cxcl1, Ccl2, Bcl2, Icam1, Ptges, *and* Tlr2 *in the pretreatment procedure (Figure 7), as well as *Icam1 *and* Il1r *in the posttreatment procedure (Figure 8). Additionally, pyrenocine A increases the mRNA levels of the anti-inflammatory cytokine *Il10 *in both the pretreatment and posttreatment protocols (Figures 7 and 8). Some genes were not amplified (data not shown), such as *Vcam1, Il12a, *and *Ccl20*, indicating that RAW264.7 macrophage cells are unable to transcribe these genes in response to LPS. Conversely, some proinflammatory genes were upregulated with the posttreatment treatment, such as *Nos2, Il1a, Cd40 *and *Il12b*, but not with the pretreatment treatment, suggesting that pretreatment procedure is able to downregulate proinflammatory genes in response to LPS (Figures 7 and 8).

## 4. Discussion

Although marine microorganisms are currently considered to be major natural sources of potently active secondary metabolites, very few natural products that have been isolated from marine microbes exhibit anti-inflammatory activity. Examples include salinamides A and B, which are depsipeptides produced by a marine streptomycete [23]; the cyclomarins, which are cyclic peptides that are also produced by a marine streptomycete [24]; lobophorins A and B, which are complex macrolides isolated from marine actinomycete cultures [25]; mangicols A and B, which are sesterterpenes isolated from the marine fungus *Fusarium heterosporum *[26]; and mycoepoxidiene, which is produced by a fungus in the genus *Diaporthe *[27]. Penstyrylpyrone, isolated from marine-derived *Penicillium *sp., was found to inhibit the synthesis of NO, PGE2, IL-1*β*, and TNF-*α* in peritoneal macrophages stimulated with LPS [28]. As microbial fermentation is a sustainable process, the search for new anti-inflammatory agents produced by marine microorganisms is an attractive strategy to facilitate the production of bioactive compounds.

To assess the anti-inflammatory profile of a specific compound, it is necessary to select a reliable approach. Our model for this *in vitro* study, specifically utilizing macrophages stimulated with the inflammatory agent LPS, allows us to evaluate the effects of immunomodulatory compounds on soluble inflammatory signaling agents and cell surface receptors as well as to elucidate the mechanism of action to identify potential new inflammatory targets. The rationale of our approach is based on the fact that macrophages, which are resident cells that are distributed throughout different tissues in the body, play a key role in the inflammatory process and have a variety of cell surface receptors that permit strong interactions with PAMPs, which are present on the pathogen surfaces, as well as with DAMPS (damage-associated molecular pattern molecules) released by cells that enter necrosis. The results described here demonstrate that pyrenocine A, a secondary metabolite produced in liquid medium by the marine-derived fungus *Penicillium paxilli* Ma(G)K, exerts potent anti-inflammatory actions both pretreatment and posttreatment on cells that are part of the innate immune response.

The anti-inflammatory activity of pyrenocine A was initially assessed by ability to modulate nitric oxide production. Treatment of macrophages with pyrenocine A using both pretreatment and posttreatment procedures in the presence of the inflammatory stimulus LPS promoted strong concentration-dependent inhibition of NO production. Activated macrophages are present in injured tissues and are major producers of inflammatory mediators such as proinflammatory cytokines and lipid mediators, which have been implicated in the exacerbation and pathogenesis of acute and chronic inflammatory diseases [29]. Various steroidal and nonsteroidal (NSAIS) drugs have mechanisms of action that involve the inhibition of proinflammatory cytokine and chemokine production, including TNF-*α*, IL-1, IL-6, and IL-8 as well as the inhibition of NO synthesis [30]. Dexamethasone is a synthetic glucocorticoid with potent anti-inflammatory effects that is used as the first-line treatment for inflammatory diseases such as acute lung injury, asthma, and rheumatoid arthritis [31]. In addition to modulating inflammatory cytokines, some drugs have the ability to increase the synthesis of anti-inflammatory cytokines such as IL-10, which is a cytokine capable of indirectly mediating the synthesis of anti-inflammatory mediators, including various surface receptors [32]. Beside dexamethasone, compounds from plants, such as ursolic acid, have a potent anti-inflammatory effect an macrophages by promoting the inhibition of TNF-*α*, IL-1, and IL-6 from macrophages in the presence of LPS as well as costimulatory molecules (CD80 and CD86) in B cells and T cells proliferation. The suppressive effect of ursolic acid is mediated, at least in lymphocytes, by the inhibition of nuclear translocation of NF-*κ*B, NF-AT, and AP-1 translocation into the nucleus. [33]. The results presented here demonstrate that pyrenocine A has both pretreatment and posttreatment anti-inflammatory effects on the synthesis of TNF-*α* and PGE2 by macrophages stimulated with LPS. Treatment with pyrenocine A did not induce increased IL-10 release, regardless of the concentration and protocol used. Although pyrenocine A did not induce statistically significant differences for IL-10 synthesis, both the pretreatment and posttreatment procedure in the presence of LPS increased the mRNA expression of IL-10. These results suggest that the kinetics of IL-10 mRNA expression and protein production may explain these contradictory results.

The recruitment of inflammatory cells to injured tissues is mediated by chemoattractants and adhesion-promoting agents such as integrins (CD11b/CD18), selectins (L-selectin), and chemokines [34]. Proinflammatory macrophages promote the recruitment and activation of neutrophils through the release of cytokines and chemokines and the expression of surface molecules such as selectins and integrins as well as the subsequent recruitment of adaptive immune response cells. Such soluble inflammatory mediators or stimuli can also modulate the expression of costimulatory molecules on dendritic cells such as B7.1 and B7.2, which are essential for T cell activation. The expression of Mac-1 on macrophages is important not only for cellular recruitment of monocytes, cell-cell contact, and activation, but also for microbial phagocytosis [35]. Our results indicate that the pretreatment and posttreatment procedure with pyrenocine A promoted the decrease of expression of the surface receptors Mac-1 and B7.1 but only induced a slight decrease in B7.2 expression. Thus, pyrenocine A inhibits cytokine synthesis and also inhibits the expression of surface receptors involved in both cell recruitment and in the activation of leucocytes. Additionally, pyrenocine A inhibits macrophage mediators that reduce the recruitment of inflammatory cells to injured tissue, thereby contributing to disease chronicity.

Soluble mediators produced by inflammatory macrophages provide the microenvironment necessary for the recruitment of inflammatory cells such as neutrophils, macrophages, and autoreactive T lymphocytes. Substantial amounts of NO and prostaglandin E2 (PGE2) are produced during the inflammatory process by iNOS and cyclooxygenase 2 (COX-2) [36]. Pyrenocine A was able to inhibit PGE2 synthesis with the pretreatment protocol but only modestly inhibited synthesis with the posttreatment approach. Similarly, *Pgtes*, one of the major enzymes involved in the synthesis of PGE2, was also inhibited when macrophages were treated with pyrenocine A using the pretreatment protocol but not the posttreatment protocol.

To assess the molecular mechanism involved in pyrenocine A anti-inflammatory actions, macrophages were stimulated with a TLR3 agonist (Poly I:C) and a TLR9 agonist (CpG-ODNs) to evaluate the MyD88-independent and MyD88-dependent signaling pathways, respectively. Since there was no suppression of nitrite production by TLR3/Poly I:C, we speculated that the suppressive effect of pyrenocine A on macrophages may be mediated by a MyD88-dependent pathway. It appears that the suppressive effects of pyrenocine A are selective, as it is able to inhibit a specific biochemical cascade and promote the suppressive actions of specific inflammatory stimuli. When we analyzed the effects of pyrenocine A on the modulation of NF*κ*B-dependent gene expression, we observed that pyrenocine A promoted the inhibition of genes such as *Cxcl1, Ccl2, Icam1*, and *Tlr2 *with the pretreatment procedure and *Icam1 *and* Il1r *with the posttreatment procedure in addition to inhibiting *Tnf *and *Ptges *mRNA expression. Conversely, pyrenocine A increased the mRNA expression of the anti-inflammatory cytokine *Il-10*, both before treatment and after treatment. Our results suggest that pyrenocine A can downmodulate the expression of additional genes that are involved in the chemotaxis of inflammatory cells (*Cxcl1, Ccl2*) and an integrin involved in recruitment and cell activation (*Icam*).

Together, our findings suggest that pyrenocine A has a potential anti-inflammatory effect on cells from innate immune response; however, the potential prophylactic and therapeutic applications of pyrenocine A need to evaluate *in vivo* inflammatory murine model.

## 5. Conclusions

In conclusion, we demonstrated that pyrenocine A, which was produced in the culture media of the marine-derived fungus *Penicillium paxilli *Ma(G)K, promotes immunosuppressive effects via the inhibition of proinflammatory mediators (TNF-*α* and PGE2) and the expression of molecules involved in cell migration (Mac-1) and T cell activation (B7.1). Our results suggest that pyrenocine A inhibits macrophage activation, probably by interfering with the MyD88-dependent pathway but not through the TRIF signaling pathway. Pyrenocine A is able to inhibit the expression of genes related to NF*κ*B activation in macrophages stimulated with LPS. Our findings indicate that pyrenocine A is novel compound with immunosuppressive properties.

## Supplementary Material

Supplemental Figure 1A: *Cell Viability Assay*. (A) Treated during 18h with pyrenocine A (3.75-0.11 *µ*M) without LPS. (B) Pretreatment procedure: cells were pretreated with different concentrations of pyrenocine A (3.75-0.11 *µ*M) for 2 h and then stimulated with LPS (1 *µ*g/mL) during 18h. (C) Post-treatment procedure: cells were stimulated with LPS (1 *µ*g/mL) for 2 h and then added pyrenocine A (3.75-0.11 *µ*M) during 18h. After the treatment, the Alamar Blue reagent was added in the same point-time with LPS and after 18 h the absorbance was read on a spectrophotometer at 570 nm, using 600 nm as a reference wavelength (normalized to the 600 nm value).
Supplemental Figure 1B. Cell death by pyrenocine A by AnexinV/PI. Cells were treated during 18h with pyrenocine A (3.75-0.94 *µ*M) without LPS. Pretreatment procedure: cells were pretreated with different concentrations of pyrenocine A (3.75-0.94 *µ*M) for 2 h and then incubated with LPS (1 *µ*g/mL) during 18h. Post- treatment procedure: cells were stimulated with LPS (1 *µ*g/mL) for 2 h and then added pyrenocine A during 18h (3.75-0.94 *µ*M). Control: cells untreated (without pyrenocine A or LPS). After the treatment the cells were harvested, stained for Annexin V/PI and acquired on FACSCanto machine and analyzed with FCS Express Software.
