# Supplementary figures and images for: Potent Anti-Inflammatory Activity of Pyrenocine A Isolated from the Marine-Derived Fungus Penicillium paxilli Ma(G)K
Source: Mediators Inflamm. 2014 Jan 19;2014:767061. doi: 10.1155/2014/767061 (PMC3916108; doi:10.1155/2014/767061)

# Supplemental data Figure 1A

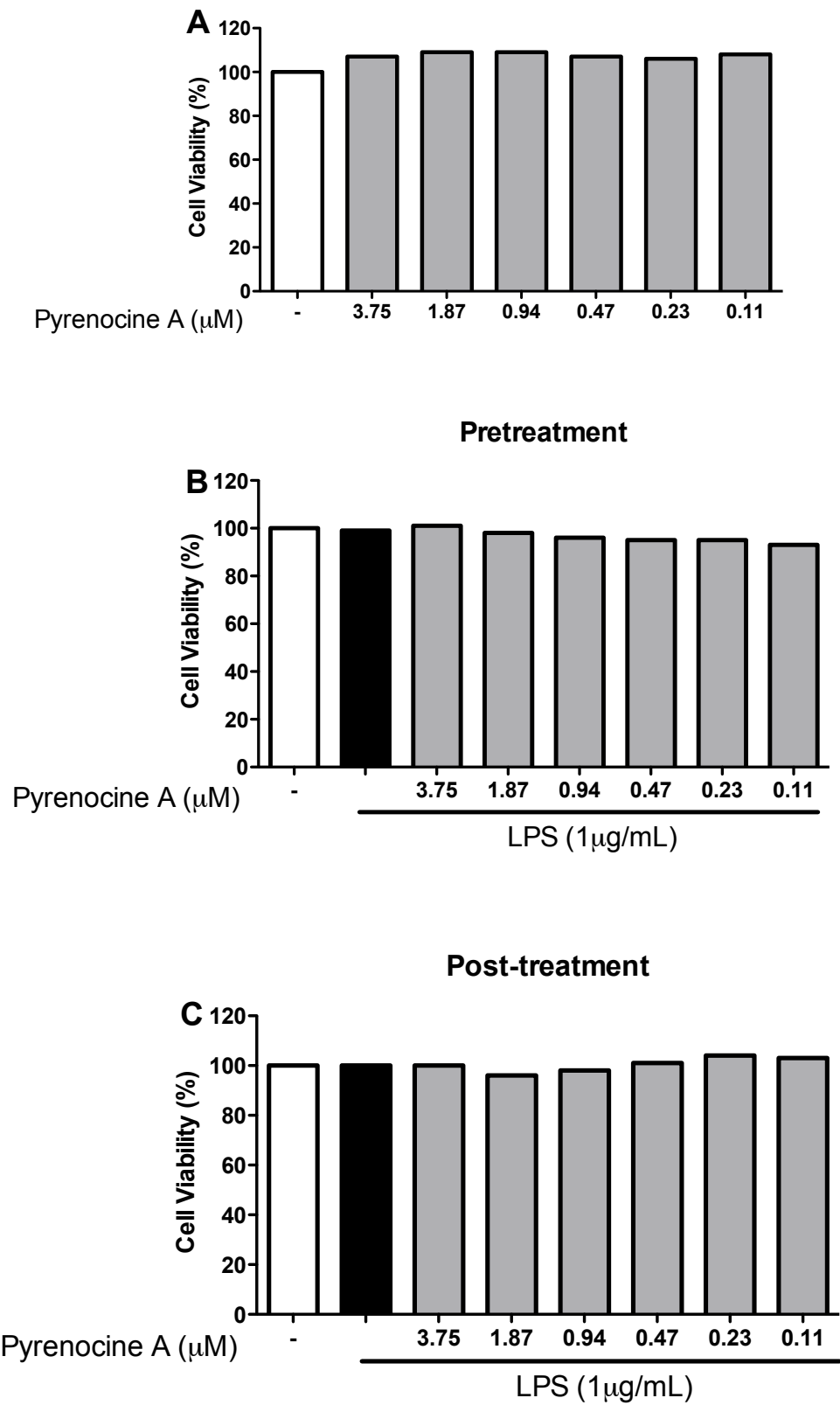

Supplement: Supplementary file 1 — Supplemental Figure 1A: Cell Viability Assay. (A) Treated during 18h with pyrenocine A (3.75-0.11 µM) without LPS. (B) Pretreatment procedure: cells were pretreated with different concentrations of pyrenocine A (3.75-0.11 µM) for 2 h and then stimulated with LPS (1 µg/mL) during 18h. (C) Post-treatment procedure: cells were stimulated with LPS (1 µg/mL) for 2 h and then added pyrenocine A (3.75-0.11 µM) during 18h. After the treatment, the Alamar Blue reagent was added in the same point-time with LPS and after 18 h the absorbance was read on a spectrophotometer at 570 nm, using 600 nm as a reference wavelength (normalized to the 600 nm value). Supplemental Figure 1B. Cell death by pyrenocine A by AnexinV/PI. Cells were treated during 18h with pyrenocine A (3.75-0.94 µM) without LPS. Pretreatment procedure: cells were pretreated with different concentrations of pyrenocine A (3.75-0.94 µM) for 2 h and then incubated with LPS (1 µg/mL) during 18h. Post- treatment procedure: cells were stimulated with LPS (1 µg/mL) for 2 h and then added pyrenocine A during 18h (3.75-0.94 µM). Control: cells untreated (without pyrenocine A or LPS). After the treatment the cells were harvested, stained for Annexin V/PI and acquired on FACSCanto machine and analyzed with FCS Express Software. [file 767061.f1.pdf]

LPS (1 $\mu$ g/mL)

Control

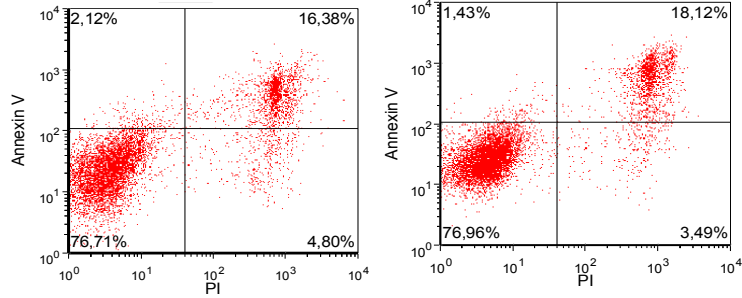

Pre-treatment

Post-treatment

3.75

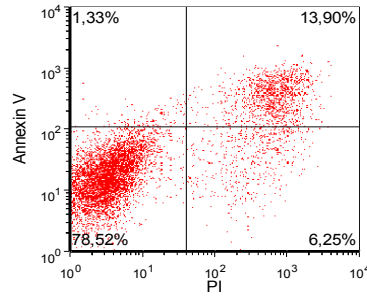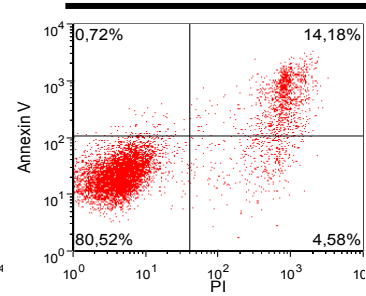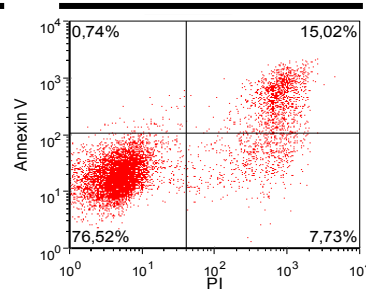

1.87

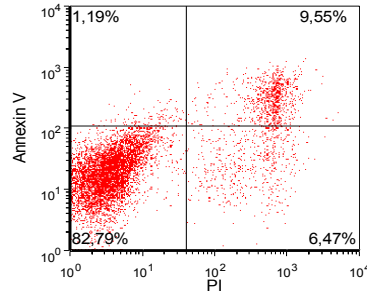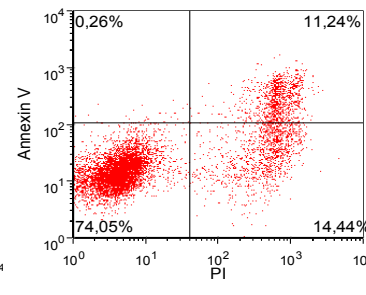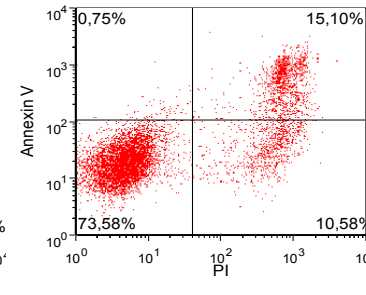

0.94

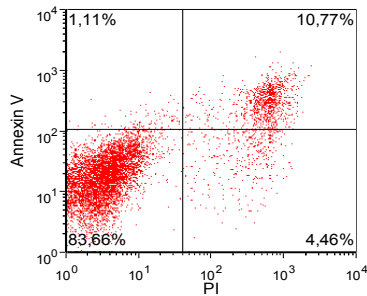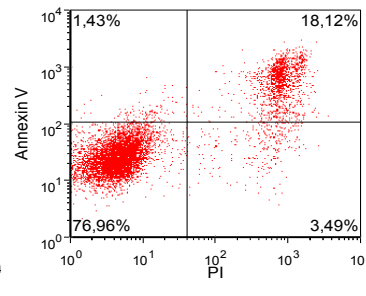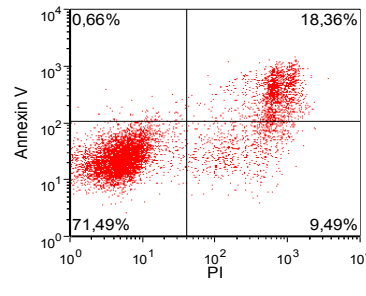

Pyrenocine A ( $\mu$ M)

Supplement: Supplementary file 2 [file 767061.f2.pdf]
